# Health and Social Care Managers' Competence in Knowledge Management Instrument: Development and Validation

**DOI:** 10.1155/nrp/9617966

**Published:** 2025-08-22

**Authors:** Eevi Karsikas, Merja Meriläinen, Kirsi Koivunen, Erika Jarva, Kristina Mikkonen, Anne Oikarinen, Maria Kääriäinen, Päivi Jounila-Ilola, Outi Kanste

**Affiliations:** ^1^Research Unit of Health Sciences and Technology, University of Oulu, Oulu, Finland; ^2^MRC Oulu, Oulu University Hospital, University of Oulu, Oulu, Finland; ^3^Oulu University of Applied Sciences, Oulu, Finland

**Keywords:** competence, health and social care organizations, instrument, knowledge management, managers, quantitative research

## Abstract

**Background:** The knowledge management (KM) competence of health and social care managers is essential for organizations to achieve and maintain competitiveness. The study aimed to develop and validate the Managers' Competence in Knowledge Management (MCKM) instrument, which assesses health and social care MCKM.

**Methods:** The study followed four phases: (1) items of the instrument were created based on a conceptual framework; (2) the content validity index was assessed by 11 experts. After that, 11 managers provided feedback on the instrument by participating in the pilot study; (3) the construct validity was examined with exploratory factor analysis (EFA); and (4) internal consistency was established with Cronbach's alpha. The data were collected from 116 Finnish managers during two separate periods in February and August 2022.

**Results:** The overall S-CVI for the MCKM instrument was 0.83. EFA revealed a five-factor model for the MCKM instrument, containing 43 items, which explained 72% of the observed variance. The factors' Cronbach's α coefficient ranged from 0.913 to 0.964. The instrument development and validation process resulted in five factors: planning competence development and cooperation, managing a culture of competence, anticipating and defining competence, developing competence, and assessing competence. Items are scored on a Likert scale of 1–5.

**Conclusions:** The instrument gave valid and reliable results in psychometric testing. It is suitable for self-assessment of competence in KM among health and social care managers.

**Practice Implications:** Examining the KM competence of health and social care managers is vital for addressing unforeseen competence and knowledge challenges.

## 1. Introduction

Knowledge management (KM) is a systematic and organized approach [1]. In health and social care settings, effective KM underpins organizational sustainability, drives continuous learning, and strengthens care quality and safety, while shaping both the motivations that enable—and the barriers that hinder—knowledge use. When implemented effectively, KM can yield a sustainable competitive advantage and deliver improvements in operational performance, productivity, service quality, financial outcomes, and patient safety [2, 3]. Previous studies have found that the possibilities for the productive use of KM remain largely untapped [3], as health and social care organizations tend to neglect KM [4], and that managers lack competence in KM [5]. Additionally, managers in healthcare often have a narrow understanding of KM [4]. This may be due to the lack of a validated assessment instrument for evaluating managers' KM competence [6]. Therefore, the aim of this study was to develop and psychometrically test the health and social care MCKM instrument.

In this study, competence is understood as encompassing managers' knowledge, skills, attitudes, values, and performance in the KM context [7, 8]. To be competent in KM, managers must be able to approach KM systematically and purposefully, using different structures, processes, methods, and information systems effectively to support management [6]. Additionally, they must act as enablers and supporters of staff professional development, serving as mentors and role models [6, 9]. This is essential because, for example, when managers support their staff, employees are motivated to reciprocate by fully immersing themselves in their work, engaging in it, and putting more passion and effort into it [10]. Moreover, managerial competence in KM includes the ability to provide constant feedback and plan for continued learning [11] and to anticipate staff competence in healthcare organizations [12]. Furthermore, adopting effective management models, such as transformative management, enhances employee competence [9], while dynamic and supportive supervision fosters informal learning motivation [13], and a playful approach strengthens employees' sense of belonging, encouraging them to take initiative and improve the work environment [14].

KM is a process that encompasses various stages, but its definition may differ depending on the specific context in which it is discussed [3, 4, 9, 15]. This is due to the lack of a universally accepted definition of the concept of KM, not only in nursing science but also in other fields [12, 16]. The SECI model is the most well-known framework for understanding how knowledge is created in organizations. It formalizes the process of knowledge creation by distinguishing between tacit and explicit knowledge and provides a practical tool for assessing knowledge creation in organizational settings [17]. In the context of healthcare, previous studies have shown that nursing managers' daily KM activities are mainly focused on ensuring smooth workflow and making decisions regarding unexpected changes [12]. In addition, its implementation is challenged by the constant need to acquire and evaluate up-to-date knowledge and apply it in a rapidly changing environment [5].

Since healthcare managers play a central, albeit sometimes invisible, role in KM, their KM competence should be systematically defined and assessed [18]. Managerial KM competence has previously been assessed using various instruments, but none of them focused on the sole measurement of managerial KM competence, and these instruments did not take into account the different phases of KM. In addition, KM is often assessed as part of a manager's overall competence [6]. For example, Karamitri et al. [19] developed the Applied Knowledge Management Assessment Instrument, which measures attitudes, emotions, cognition, intention, and behavior and identifies the motivators and barriers for employees in KM. Lunden et al. [18] measured nurses' perceptions of organizational support in enhancing competence and evidence-based practice.

It is essential to develop a new MCKM instrument based on the latest research to fill this gap. The uniqueness and importance of this instrument lies in its ability to recognize KM competence as a distinct subcategory of management, an aspect that has been overlooked in previous literature [20, 21]. It treats KM as a comprehensive framework, a perspective that previous KM instruments have not addressed. For example, the KM process approach contributes to the relevance and value of the instrument in current research [22]. In addition, the specificities of both the social and health care sectors have been considered in the development of the instrument, which enriches the subject and provides a deeper understanding of the approach to KM.

## 2. Methods

### 2.1. Study Design

**Figure 1:**
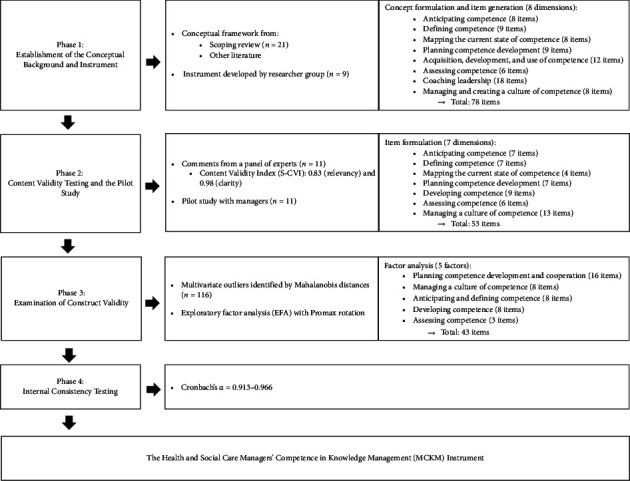
Developmental phases of the managers' competence of knowledge management instrument.

The study consists of four phases: (1) establishment of the conceptual framework and items of the instrument; (2) content validity testing and pilot study; (3) examination of construct validity; and (4) internal consistency testing (Figure 1). The COnsensus-based Standards for the selection of health status Measurement INstruments (COSMIN) checklist was followed during the entire process to enhance the quality of the study [23].

### 2.2. Phase 1: Establishment of the Conceptual Framework and Instrument

The first phase in constructing the instrument was to develop a large pool of possible items [24]. This step is crucial because once the final instrument has been created, there is no way to compensate for items that were unintentionally omitted [25]. Typically, this initial pool contains 50% more items than the final instrument [26]. In this MCKM instrument, item development was based on a scoping review (blinded for review). In addition, the framework was complemented by a review of other literature and research [1, 2, 2, 7, 12, 18, 19]. The first phase also included the definition and operationalization of the concepts [27]. Next, the items were formulated by the research team (*n* = 9) and modified to measure self-reported managerial KM competence (“I can…”). The research team included instrument development experts, nursing and rehabilitation experts, management experts, and management teachers. From the beginning of the study, the items were designed with clarity, jargon, reading level, length, double negatives, and double-barreled questions in mind [24, 25].

### 2.3. Phase 2: Content Validity Testing and the Pilot Study

During the second phase, the instrument's content validity was evaluated with the Content Validity Index (CVI) using one round of assessment [28]. To determine content validity, 11 experts reviewed the items for relevance and clarity. The experts were recruited using purposive sampling and included instrument development experts, KM experts, managers at different organizational levels, advanced practice nursing and rehabilitation experts, and management teachers and students. The educational levels of the experts ranged from a university of applied sciences to doctoral degrees. The responses were used to calculate the individual item CVI (I-CVI) and the total score averages (S-CVI). The cutoff for an acceptable score was ≤ 0.78 for the I-CVI and 0.70–0.80 for the S-CVI [24].

The panel assessment was followed by a pilot study of the instrument. The pilot study was conducted to determine the comprehensibility, readability, and wording of the background questions and items of the instrument, as well as the suitability of the response options, the structure of the instrument, and the duration of the response period [27, 29]. Managers who were or had been working in a health or social care organization (*n* = 11) were invited to pilot the instrument through purposive sampling. This selection process ensured that the participants invited to partake in the pilot study represented the intended population for which the instrument had been designed [25]. Purposive sampling was used for the content validity test (September 2021) and the pilot study (October 2021).

### 2.4. Phase 3: Examination of Construct Validity

At the beginning of the third phase, multivariate outliers were identified and managed [27]. In this study, they were detected by calculating Mahalanobis distances with *p* values of below 0.001 [30]. Next, the examination of psychometric properties was conducted using exploratory factor analysis (EFA), which assumes no prior hypotheses about the conditionality of a set of items [24]. The EFA employed principal axis factoring with a Promax rotation. The sample adequacy and the appropriateness of the factor model were assessed using the Kaiser–Meyer–Olkin test (KMO; > 0.60) and Bartlett's test (*p* value < 0.05). The cutoff for item loading to a factor was set at 0.30 or higher to improve accuracy, and in this study, the cutoff was set at 0.40 [24, 31]. The analyses were performed using IBM SPSS Statistics (Version 28.0). Tests of construct validity were derived from data collected during the period February and August 2022.

### 2.5. Phase 4: Internal Consistency Testing

In phase four, the internal consistency of the instrument was calculated using Cronbach's *α*, which indicates an estimate of the extent to which different dimensions (factors) of an instrument reliably measure the critical attribute [24]. All items of the new instrument have a threshold of 0.70, which is characteristic of a usable measure [26]. Internal consistency tests were derived from data collected during the period February–August 2022.

### 2.6. Sampling and Data Collection

The data were collected by conducting an electronic survey (Webropol software) in six primary and special medical health and social care organizations in Finland during two separate periods in February and August 2022. In February 2022, an email was sent to all 235 eligible managers in a university hospital and two healthcare organizations, resulting in an 18% response rate (42 managers). To increase the sample size, in August 2022, data collection expanded to three more hospital districts, with all 414 eligible managers contacted, resulting in an 18% response rate (74 managers).

A total of 649 participants were invited in the study, with the aim of achieving at least 3–5 respondents per item (*n* = 120 or more) [24]. All managers who worked in social, health, and rehabilitation units or organizations received an e-mail invitation to participate in the study. In Finland, talk about health and social care managers because health and social care services have been integrated into a single entity in the sector, with administration and cross-sectoral management centralized under the same officials [32]. According to a recent report, the workforce is divided into two main sectors: approximately 60% in health care and 40% in social care [33].

The e-mail described the study and provided a link to the consent form and anonymous survey. An electronic survey was chosen as a cost-effective and efficient method for recruiting participants and collecting data since all employees had email addresses [34]. One reminder email was sent to managers after 2 weeks. The electronic survey consisted of two parts: The first part included 15 background questions, seven of which are described in Table 1, and the second part was the MCKM instrument. Managers rated their competence using a 5-point Likert scale on the MCKM instrument: 1 = *poor*, 2 = *fair*, 3 = *good*, 4 = *very good*, 5 = *excellent*.

### 2.7. Ethical Considerations

The trial adhered to the ethical principles of the Declaration of Helsinki [35]. Research permission was obtained from each organization. Research ethics committee approval was not required because the study did not involve minors, clinical trials, or direct or indirect physical or physiological harm to participants [36]. Participants received an electronic invitation containing details about the study's purpose and objectives, assurances of confidentiality, the right to withdraw without consequence, and contact information for the research group for further inquiries. Informed consent was obtained from each participant in accordance with the EU General Data Protection Regulations [37], and participants completed the survey anonymously. The research data were treated confidentially, as required by the GDPR [43], and stored securely in password-protected files. Data will be destroyed 10 years after data collection.

## 3. Results

### 3.1. Sample Characteristics

There were 116 responses in total, with a response rate of 17.9%. The majority of the participants were female (82.7%) and aged 48–57 years (35.3%). The most common educational backgrounds reported were a master's degree from a university (34.5%) and a bachelor's degree from a university of applied sciences (34.5%). Almost all managers worked in management full time (89.7%), and the mean work experience as a manager in health and social care was 11.9 years. The majority of respondents (69.8%) worked in healthcare, while 21.6% were employed in social care (Table 1).

### 3.2. Development and Psychometric Testing of the MCKM

The results are presented according to four phases: (1) establishment of the conceptual framework and items of the instrument; (2) content validity testing and pilot study; (3) an examination of construct validity; and (4) internal consistency testing (Figure 1).

#### 3.2.1. Phase 1: Establishment of the Conceptual Framework and Instrument

The first step in the development of MCKM involved a scoping review (blinded for review) to identify the dimensions of KM competence that health and social care managers have and need as well as to identify instruments previously used to assess managerial KM competence. Based on the dimensions and instruments identified in the review and a survey of previous literature [9, 18, 19], over 120 preliminary items were identified by the research group (*n* = 9). The research group then edited the preliminary items together, resulting in 78 items organized under KM dimensions. The instrument had a total of eight dimensions, with the first six dimensions encompassing a KM process: anticipating competence (8 items), defining competence (9 items), mapping the current state of competence (8 items), planning competence development (9 items), acquisition, development, and utilization of competence (12 items), and assessing competence (6 items; [15]). The remaining two dimensions focused on management: coaching leadership (18 items) and managing and creating a culture of competence (8 items). The number of items per dimension ranged from 6 to 18.

#### 3.2.2. Phase 2: Content Validity Testing and the Pilot Study

The content validity of the MCKM was tested over one round of expert evaluation. In the evaluation panel, experts rated the relevance of each item using a 4-point ordinal rating instrument (1 = *irrelevant, should be deleted*; 2 = *relevant but needs a lot of change*; 3 = *relevant but needs minor changes*; 4 = *completely relevant*). The I-CVI was calculated for each of the 78 items by dividing the number of experts who scored an item's relevance as 3 or 4 by the total number of experts. The average I-CVI of the items was 0.78, which was deemed adequate [24]. Additionally, the panel experts evaluated the clarity of each item using a 4-point ordinal rating instrument (1 = *very unclear, should be deleted*; 2 = *fairly clear but needs a lot of change*; 3 = *clear but needs small changes*; 4 = *very clear*). The I-CVI was calculated as described earlier, with the same cutoff value applied. Only one item in the dimension of defining competence was removed due to a low I-CVI value (0.64).

Experts also provided helpful verbal recommendations related to the relevancy and clarity of the remaining 77 items [27]. For example, many participants reported that instrument development was necessary and comprehensive and that it additionally measured the right things. Based on this feedback, dimensions and items were deleted or modified. The most common reason for removing items was that the items were too similar or difficult to understand. Additionally, two management dimensions were combined. Finally, the instrument consisted of 53 items categorized into seven dimensions. The S-CVI value for the instrument was calculated by dividing the sum of the I-CVI values by the number of items. The S-CVI value was above the limit indicated by the literature (0.70–0.80) throughout the editing process and eventually reached 0.83 for relevance and 0.98 for clarity [24]. Finally, a pilot study of the instrument was conducted with 11 managers in a university hospital, and the results indicated that the instrument had an appropriate structure and number of items. None of the items needed to be modified based on feedback from the participants in the pilot study.

#### 3.2.3. Phase 3: Examination of Construct Validity

Psychometric properties were tested using data from a sample of managers (*n* = 116). There were no missing values in the survey, since the survey included only mandatory items. First, multivariate outliers were identified by calculating Mahalanobis distances with a threshold *p* value of < 0.01 [27, 30]. No multivariate outliers were detected. Next, an EFA with Promax rotation was conducted to identify the factors of the remaining 53 items of the instrument. KMO and BTS were performed to examine sample adequacy. Findings of the preliminary factor analysis showed a KMO value of 0.934 (BTS, *p* < 0.000), which was above the desired value of 0.8 and indicates the adequacy of the instrument's items for factor analysis [27, 31]. The decision on the number of factors to be extracted was based on eigenvalues of 1 and above and scree plot results. To eliminate cross-loading and obtain robust factors, small coefficients with absolute values below 0.40 were deleted [31]. In addition, the commonality values were calculated, which ranged from 0.468 to 0.857.

Finally, a table was created displaying the five factors of the instrument. The five-factor loading explained 72.281% of the cumulative percentage of the total variance. The first factor, *planning competence development and cooperation* (16 items), explained 53.458% of the total variance; the second factor, *managing a culture of competence* (8 items), explained 8.001% of the variance; the third factor, *anticipating and defining competence* (8 items), explained 4.569% of the variance; the fourth factor, *developing competence* (8 items), explained 3.280% of the variance; and the fifth factor, *assessing competence* (3 items), explained 2.972% of the variance (Table 2).

#### 3.2.4. Phase 4: Internal Consistency Testing

The reliability of the 43 items was assessed using Cronbach's α. The internal consistency of the factors ranged from 0.913 to 0.966, and the overall instrument α was 0.920 [26].

## 4. Discussion

This study aimed to develop and validate a new instrument that measures the KM competence of health and social care managers. The instrument measures managerial KM competence based on five dimensions: planning competence development and cooperation, managing a culture of competence, anticipating and defining competence, developing competence, and assessing competence. The importance of measuring the KM competence of health and social care managers lies in the fact that their competence is crucial in implementing KM strategies in health and social care organizations [39]. Effective KM is closely linked to improved organizational performance—better KM leads to greater effectiveness [3]. Although management competence instruments do exist, measuring managerial KM competence is currently a challenge, as KM is not yet recognized as a separate area of management in the health and social care environment [40].

To the best of our knowledge, this is the first study to provide a validated instrument for measuring the KM competence of health and social care managers. Although various instruments have been developed in the past to measure KM in health and social care organizations, this instrument examines the phenomenon from the perspective of managers' self-reported KM competence. The developed MCKM instrument makes a novel contribution to the study of KM in health and social care organizations. It examines managerial KM competence from a new and comprehensive perspective, viewing the KM process as a broad and integrated entity that spans all levels of the organization [3, 4, 9]. Moreover, the instrument acknowledges both the social and technical dimensions essential for effectively managing knowledge in health and social care settings [3]. Thus, the MCKM instrument can be used to examine KM as a process or to focus on a specific dimension (=factor) of the KM process in health and social care organizations.

Based on our findings, the first factor is *planning competence development and cooperation*, which includes the managers' ability to strategically plan competence development using forecasting information and to utilize collaboration in all dimensions of KM. Previous studies have shown that current succession planning strategies in healthcare organizations are inadequate or completely lacking [4]. In addition, managers have expressed concerns about the need for improvement in how they utilize and leverage networks and cooperation for KM [22]. In this MCKM instrument, compared to the instrument developed by Karamitri et al. [19], managers' cooperation competence has been expanded to include cooperation both inside and outside the organizations. This expansion is in line with the study findings of Karsikas et al. [22], where they investigate the factors that influence managers' KM competence.

The second factor is *managing a culture of competence*, which is managers' ability to create an environment that supports competence development, for example, by supporting the professional growth of staff, motivating them to participate in continuous education, and encouraging them to try new methods of competence development. In addition, the study identified managers' competence in rewarding staff as an area that has been little researched, even though it has been found that various reward systems encourage staff to work more efficiently and can be a factor in choosing a particular job [41]. Previous research has shown that a culture in which, for example, knowledge sharing takes place has a positive effect on employees' work engagement [10]. Moreover, when employees can make decisions and manage their work, they remain more engaged and strive to improve [14]. However, creating a supportive work environment where staff feel encouraged to share knowledge remains one of the most challenging tasks for managers [42]. This difficulty is compounded by the cultural dichotomy within healthcare organizations [4] and the lack of organizational support [9], both of which have been identified as significant limiting factors. According to Lunden et al. [18], the role of managers is to build a knowledge-enhancing culture by supporting, challenging, and inspiring staff.

The third factor, *anticipating and defining competence*, includes the manager's competence to determine future competence needs, anticipate development needs, and determine the staff's competences. From the previous studies by Karsikas et al. [22] and Lunden et al. [18], health and social care managers need to have predictive competence to anticipate the short- and long-term training needs of their staff. In addition, they should be able to establish a mission, vision, strategy, and goals in KM and define development areas in staff competence [9].

The fourth factor, *developing competence*, refers to managers' ability to enhance staff proficiency through various approaches. This includes staff competence development, expanding work and responsibility tasks, engaging in study and training, leveraging existing personnel competencies, tapping into tacit knowledge, and recruiting personnel to ensure the unit's competence. In addition to formal training, the provision of nonformal learning opportunities is crucial, as such methods have been linked to improved knowledge sharing in organizations [13]. According to Karsikas et al. [6, 22], managers' versatile utilization and effective management of competence development practices and methods, such as job rotation, coaching, and mentoring, significantly impact managers' competence in KM within health and social care environments.

Findings indicated that the fifth factor, *assessing competence*, includes managers' ability to assess factors that both promote and hinder staff competence development, as well as staff commitment to competence development. This complements previous research, as systematic literature reviews have not recognized competence assessment or the managerial role in KM, despite its critical significance [3]. Effective competence assessment enables managers to identify strengths and weaknesses within their staff [18]. However, there were few formal tools and processes that met the needs of managers and helped them make informed decisions to assess staff competences in their healthcare organizations [4, 6].

In this study, psychometric tests showed that the MCKM instrument, at its current stage of development, is a reliable and valid measure of health and social care managers' KM competence. The psychometric testing presented in this study was based on appropriate research methods and the instrument development process. The conceptual framework was used to support the content validity of the MCKM instrument. During the development of the MCKM instrument, information obtained from experts was used to modify, delete, and edit instrument items. The selection of which items will be included in the final instrument is always crucial, as poorly developed instruments can cause researchers to draw invalid conclusions about the studied phenomena [26].

The objective of this instrument is to gauge the levels of health and social care managers' KM self-assessed competence, determine future training needs, and identify development target levels. By focusing on managers' KM competence, the instrument supports management development within public social and health care organizations. It addresses a recognized research gap, as KM has been rarely studied in this sector [43]. The instrument also helps managers understand the specific competencies required of them throughout the KM process. Additionally, KM is a global phenomenon. Therefore, the use of the MCKM instrument in the future is not limited to Finland. It can be translated into other languages, validated, and used internationally in various health and social organizations because the conceptual framework of the developed instrument was based on a systematic scoping review (blinded for review).

### 4.1. Limitations

There are several limitations to this study.

First, a relatively low overall response rate of 17.9% suggests that the respondents may not be fully representative of social and health care organization managers. The relatively low response rate was influenced, among other factors, by the busy and overloaded schedule of managers during the data collection period, attributed to the COVID-19 pandemic and a staff strike. In addition, the low response rate may have produced a selection bias, meaning that only managers who were more interested and competent in their KM competence participated in this study. Second, the reliability of the results may be affected by using subjective self-assessments by social and health care managers. Such assessments are more prone to bias, emotional reasoning, and social desirability effects, where respondents may overestimate desirable and underestimate undesirable traits [44].

Third, the sample size did not fully meet the minimum EFA requirements [31]. The survey response rate of 17.9% was relatively low, and a larger sample might have improved the generalizability of the results [26]. The resulting five-factor solution is therefore preliminary and suggestive. However, the study sample was, in many ways, representative of health and social care managers (Table 1), and this representativeness should instill confidence in the factor analysis results. In addition, we conducted a sensitivity analysis where we considered the statements of one or more factors and repeated the same analysis as we did with the full data.

Fourth, because the survey was anonymous, we had no way to track who completed the electronic survey or why 211 people began to fill in the instrument but did not complete it. One possible reason for suspending the instrument was the hurry of managers, although the completion time mean was short (12.9 min). Fifth, data collection for both stages was conducted for only 4 weeks and included just one reminder email, which may have contributed to the low response rate. Sixthly, the MCKM was developed and tested in the Finnish health and social care context. Thus, cultural equivalence and linguistic differences must be considered if the instrument is to be applied in another country or environment [45].

Finally, the sample of managers participating in the study was recruited from one hospital district of Finland, which limits the generalizability of the results to other populations. Therefore, further studies with larger, more diverse samples from different countries are recommended to establish the instrument's generalizability and validity.

### 4.2. Recommendations for Further Research

Further development of the MCKM instrument will be important. Future research should include larger data sets to test the five factors further. It is also recommended that the EFA be administered to different subsamples. In addition to translation and validation, it is essential to consider the identification of new research evidence and the inclusion of relevant dimensions in the MCKM instrument. The utilization of the developed instrument in longitudinal studies would also be justified, as it would allow for the assessment of managers' KM competence over an extended period within health and social care organizations. Furthermore, the MCKM could also be used by managers to recruit new managers and for development discussions on managers' career development and support needs.

## 5. Conclusions

This study describes the process of developing and validating an instrument that covers the five dimensions of the KM process. The instrument helps to understand the importance of KM for organizational success and considers the anticipation, definition, planning, development, and assessment of competence, the culture of competence, and cooperation. Through systematic development and psychometric testing, this study contributes knowledge via the introduction of a new MCKM instrument. It is clear that competent managers who are aware of their competence are better able to lead the competence of professionals and organizations. Recognition of the importance of competence in KM as a part of health and social care managerial competence and its assessment offers possibilities for developing interventions and educational programs for the enhancement of KM in health and social care organizations.

## Figures and Tables

**Table 1:** Participant characteristics (*n* = 116).

Characteristics	*n*	%	Mean (SD)	Range
Gender				
Female	96	82.7		
Male	19	16.4		
Other	1	0.9		
Age (years)			50.2 (8.8)	31–65
< 37	10	8.6		
37–47	35	30.2		
48–57	41	35.3		
≥ 58	30	25.9		
Highest education				
Doctoral degree	5	4.2		
Master's degree (university)	40	34.5		
Master's degree (university of applied science)	30	25.9		
Bachelor's degree (university)	1	0.9		
Bachelor's degree (university of applied science)	40	34.5		
Working experience in a health and social care management position (years)			11.9 (9.2)	0.4–41.7
< 10	57	49.2		
10–19	40	34.5		
20–29	12	10.3		
≥ 30	7	6.0		
Employment status				
Full time	104	89.7		
Part time	12	10.3		
Area of work				
Health care	81	69.8		
Social care	25	21.6		
Rehabilitation services	6	5.2		
Other (e.g., support service)	4	3.4		
Health and social care setting				
Public specialized medical care (e.g., university hospital)	55	47.5		
Public primary health and social care (e.g., health clinic)	49	42.2		
Other (e.g., prehospital care and disability services)	12	10.3		

*Note:* Source: Authors' own work.

**Table 2:** Exploratory factor analysis of the MCKM instrument (*n* = 116).

Item	Factor loading				
	1	2	3	4	5
*Factor 1: Planning competence development and cooperation*					
1. I can plan the development of staff competence using cooperation networks (e.g., training organizations).	0.954				
2. I can use cooperation networks to develop staff competence (e.g., training organizations and regional authorities).	0.858				
3. I can use cooperation networks to map the current state of competencies of staff (e.g., training organizations and regional authorities).	0.847				
4. I can use cooperation networks to define staff competencies (e.g., training organizations).	0.847				
5. I can use cooperation networks to assess staff competencies (e.g., training organizations and regional authorities).	0.844				
6. I can make a staff development plan.	0.836				
7. I can plan the development of staff competencies in a strategic way.	0.831				
8. I can use foresight data to plan staff development.	0.783				
9. I can define my responsibilities in planning the development of staff competencies.	0.759				
10. I can assess staff competencies based on indicators and metrics.	0.655				
11. I can use digital solutions for competence development (e.g., applications and systems).	,623				
12. I can choose methods and practices for developing staff competencies.	0.576				
13. I can choose the priority areas for staff development.	0.550				
14. I can develop my competence in knowledge management.	0.532				
15. I can utilize cooperation networks to anticipate staff competencies (e.g., training organizations and regional authorities).	0.502				
16. I can take responsibility for knowledge management.	0.422				

*Factor 2: Managing a culture of competence*					
17. I can give constructive feedback to my staff.		0.948			
18. I can promote the adoption of good practices.		0.847			
19. I can reward staff for developing their competence.		0.800			
20. I can support the professional growth of staff.		0.791			
21. I can support staff in open communication.		0.773			
22. I can motivate staff to participate in training.		0.756			
23. I can support staff in sharing knowledge.		0.650			
24. I can inspire staff to try new methods to develop their competence.		0.544			

*Factor 3: Anticipating and defining competence*					
25. I can determine the complementary competence of staff (e.g., secretarial services and IT).			0.782		
26. I can determine the deep knowledge of staff (e.g., specialized knowledge).			0.772		
27. I can determine the core competencies of staff (competencies that the organization considers essential for its own or its staff's activities).			0.697		
28. I can identify the tacit knowledge that staff possess (nonverbal knowledge or competence that is accumulated by people through experience and that is difficult to express in words and numbers).			0.697		
29. I can identify the future competence needs of staff based on the strategic objectives of the organization.			0.574		
30. I can determine my responsibility for defining staff competence.			0.499		
31. I can identify what competence staff lack.			0.496		
32. I can predict which staff competencies require the most development.			0.435		

*Factor 4: Developing competence*					
33. I can use competence management methods to develop staff competencies (e.g., development interviews and competence surveys).				0.769	
34. I can use staff competence to develop the unit's competence.				0.766	
35. I can utilize guidance methods to develop staff competence (e.g., induction and mentoring).				0.765	
36. I can use collaboration to develop staff competence (e.g., teamwork and working in pairs).				0.633	
37. I can use the extension of tasks and responsibilities to build up staff competence (e.g., substitutions and work rotation).				0.628	
38. I can use studying and training in the development of staff competence (e.g., continuing education and degrees).				0.598	
39. I can recruit staff to ensure the unit's competence.				0.492	
40. I can use tacit knowledge to develop staff competence (nonverbal knowledge or competence that is accumulated by people through experience and that is difficult to express in words and numbers).				0.413	

*Factor 5: Assessing competence*					
41. I can assess the factors that inhibit the development of staff competencies.					0.608
42. I can assess the factors that promote the development of staff competence.					0.575
43. I can assess staff commitment to competence development.					0.549
Eigenvalue	22.987	3.440	1.965	1.411	1.278
Percentage of variance explained	53.458	8.001	4.569	3.280	2.972
Total percentage of variance explained					72.281
Cronbach's alpha of factors	0.966	0.935	0.921	0.936	0.913

*Note:* Extraction method: principal axis factoring with Promax rotation; cutoff value ≥ 0.40 for factor loadings. Source: Authors' own work.

## Data Availability

Data are not shared outside of the research group.

## Supporting Information

The Consensus-based Standards for the Selection of Health Status Measurement Instruments (COSMIN) checklist was adhered to throughout the process, with details provided in the supporting information.
