# Supplementary material for: Health and Social Care Managers' Competence in Knowledge Management Instrument: Development and Validation
Source: Nurs Res Pract. 2025 Aug 22;2025:9617966. doi: 10.1155/nrp/9617966 (PMC12396907; doi:10.1155/nrp/9617966)
Supplement: Supporting Information — Additional supporting information can be found online in the Supporting Information section. [file 9617966.f1.docx]

**Supplementary material:** The COnsensus-based Standards for the selection of health status Measurement INstruments (COSMIN) guideline checklist.

| **Report section:** | **Item description** | **Page nro.** |
| --- | --- | --- |
| **Title** | | |
| The name of instrument | The name of the instrument (Managers' competence in knowledge management (MCKM)) is included in the title. | 1 |
| Measurement property | The psychometric testing is included in the title. | 1 |
| Study sample | The study sample (managers) is included in the title. | 1 |
| **Abstract** | | |
| The name of instrument | The name of the instrument (MCKM) and the type of instrument (self-evaluation) is reported. | 3 |
| Measurement property | The validity and reliability of the instrument development is described. | 3 |
| Design | The study design (a cross sectional study) are described. | 3 |
| Sample | The sample size (n = 116) and geographic location (Finland) are described. | 3 |
| Methods | The instrument went through four phases: (1) creation of the items; (2) assessment of face and content validity index, and implementation of a pilot study; (3) examination of psychometric properties; and (4) establishment of internal consistency. | 3 |
| Result | The content validity index, exploratory factor analysis and Cronbach`s alpha values were described. | 3 |
| Discussion/ Conclusion | The level of psychometric properties of the instrument was evaluated, and the instrument's usability was described in the context of existing evidence. | 3 |
| **Introduction and background** | | |
| Name and describe the PROM of interest | In the introduction, the name of the instrument is mentioned, and its intended use is described. In the methods section, the total instrument is described in greater detail. | 3-5 |
| Target population | The instrument is developed to assess the competence of health and social care managers in knowledge management. | 3-5 |
| Citation of the original development of instrument | Citations are provided for the theoretical framework, which is based on scoping literature review, modification of the validated MCKM, and generation of new items | 3-5 |
| State of knowledge and rationale | Previously designed instruments for preceptor evaluation and for preceptor self-evaluation were described. | 3-5 |
| Definitions | Definitions of all used terms are presented. | 3-5 |
| Objectives and Hypothesis | The objectives of the study were presented, and no hypothesis was set for this study. | 3-5 |
| **Methods** | | |
| Study design | Four phases (Figure 1):   1. Phase 1: Establishment of the theoretical background and instrument (Scoping review) 2. Phase 2: Face and content validity testing (content validity index (CVI)), and pilot study 3. Phase 3: Examination of psychometric properties (exploratory factor analysis) 4. Phase 4: Internal consistency testing (Cronbach's alpha) | 5 |
| Participants | Four phases (Figure 1):   1. Phase 1: Research team (n = 9) 2. Phase 2: Expert panel (n = 11) and pilot study (n =11) 3. Phase 3 and Phase 4: Manager from six health and social care organizations (N = 649; n = 116) | 6 |
| Instrument administration | The data were collected during the summer and autumn of 2022 using Webropol -software. | 6 |
| Data collection procedure | The data collection time frame and the number of reminders were described. | 6 |
| Power /sample size calculation | Study sample size was estimated by counting 3-5 participants per instrument item. | 6 |
| Statistical analyses | IBM SPSS Statistics (version 28.0) was used to test the structural validity of the instrument, and the Kaiser-Mayer-Olkin (KMO) test and Bartlett's test of Sphericity (BTS) were utilized to evaluate sampling adequacy. | 6 |
| Missing data | It was reported that there was no missing data. | 6 |
| Post hoc analysis | Not applicable in this study | - |
| **Results** | | |
| Missing data | There was no missing data | - |
| Participants characteristics | The participant characteristics included 15 background questions, and seven of these questions are reported in Table 1. | 7-8 |
| Sample size | The sample size for each background variable is described in frequencies and percentages in detail in Table 1. | 7-8 |
| **Discussion** | | |
| MP evidence | Measurement properties are described in the results section and in greater detail in a Table 2. | 8-10 |
| Practical relevance | Instrument`s practical relevance is discussed from perspectives of education and management. | 8-10 |
| Strength and limitations | The strengths and limitations of the study are extensively discussed. | 8-10 |
| Generality | Generalizability issues are discussed in the limitation section. The sample of managers was homogeneous, which limits the generalizability of the results to a wider population and recommends further national and international studies. | 8-10 |
| Instrument changes | Psychometric were discussed to be adequate. It was discussed that further validation of the instrument using different datasets is needed, such as cultural validation in different countries and environments, to enhance the generalizability of the results. | 8-10 |
| Future research | Future research recommendation are further national and international studies, which could validation of instrument with different and larger data sets. | 8-10 |
| **Conclusion** | | |
| Conclusion | The overall conclusion is that the MCKM is a valid and reliable instrument. | 11 |
| **Other information** | | |
| Conflict of interest | Conflict of interest are discussed in required Title page at this point also. | 1 |
| **Content Validity** | | |
| Relevace | An expert panel consisting of 11 experts was asked to evaluate the relevance of each item. | 5-8 |
| Comprehensiveness | An expert panel consisting of 11 experts was asked to evaluate the relevance and clarity of each item and provide additional comment. | 5-8 |
| Comprehensibility | An expert panel (n =11) were asked to evaluate the clarity of each items, and in the pilot (n = 11) evaluated to determine the comprehensibility. | 5-8 |
| Relevance results | One item was deleted due to low score of relevance and clarity. | 5-8 |
| Response options and recall period | Eleven managers were asked to participate in a pilot test to assess the suitability of the response options, the structure of the instrument, and the length of the response period. | 5-8 |
| Comprehensiveness results | One item was deleted due to low score of relevance and clarity. | 5-8 |
| Comprehensibility results | It is described that based on the feedback from the participants, none of the items needed to be modified. | 5-8 |
| **Structural validity** | | |
| Factor analyses: classical test theory | An exploratory factor analysis (EFA) was conducted because no clear a priori hypothesis was available. The cut-off loading for EFA was set and the number of factors was determined based on the theoretical framework and by counting the number of eigenvalues. EFA using principal axis factoring and Promax variation was performed, and the five resulting factors were described with the percentage of total variance explained (Table 2). | 5, 7 |
| Item Response theory (IRT) analyses | Not applicable in this study | - |
| **Internal consistency** | | |
| Unit of measurement | Cronbach’s alpha was used to assess internal consistency for each factor and for entire instrument (Table 2). | 5,7 |
| Continuous scores | Cronbach's alpha values were calculated for each of the seven factors (Table 2). | 7 |
| Dichotomous scores | Not applicable in this study | - |
| **Reliability** | | |
| PROM administrations | Cronbach's alpha was used to assess the internal consistency of each factor and the entire instrument. (Table 2). | 5,7 |
| Statistical analyses | Cronbach's alpha was used to assess the internal consistency of each factor and the entire instrument. (Table 2). | 5,7 |
| Methods to improve reliability | Cronbach's alpha was used to assess the internal consistency of each factor and the entire instrument. (Table 2). | 5,7 |
| **Criterion validity** | | |
| Criterion | It was not done in this study, because there were no instruments to be used as criterion. | - |
| Continuous scores | It was not done in this study, because there were no instruments to be used as criterion. | - |
| Categorical scores | It was not done in this study, because there were no instruments to be used as criterion. | - |
